# Translation of obstructive sleep apnea pathophysiology and phenotypes to personalized treatment: a narrative review

**DOI:** 10.3389/fneur.2023.1239016

**Published:** 2023-08-24

**Authors:** Walter T. McNicholas, Henri Korkalainen

**Affiliations:** ^1^School of Medicine and the Conway Research Institute, University College Dublin, Dublin, Ireland; ^2^Department of Respiratory and Sleep Medicine, St. Vincent’s Hospital Group, Dublin, Ireland; ^3^Department of Technical Physics, University of Eastern Finland, Kuopio, Finland; ^4^Diagnostic Imaging Center, Kuopio University Hospital, Kuopio, Finland

**Keywords:** obstructive sleep apnea, pathophysiology, phenotypes, precision medicine, personalized treatment

## Abstract

Obstructive Sleep Apnea (OSA) arises due to periodic blockage of the upper airway (UA) during sleep, as negative pressure generated during inspiration overcomes the force exerted by the UA dilator muscles to maintain patency. This imbalance is primarily seen in individuals with a narrowed UA, attributable to factors such as inherent craniofacial anatomy, neck fat accumulation, and rostral fluid shifts in the supine posture. Sleep-induced attenuation of UA dilating muscle responsiveness, respiratory instability, and high loop gain further exacerbate UA obstruction. The widespread comorbidity profile of OSA, encompassing cardiovascular, metabolic, and neuropsychiatric domains, suggests complex bidirectional relationships with conditions like heart failure, stroke, and metabolic syndrome. Recent advances have delineated distinct OSA phenotypes beyond mere obstruction frequency, showing links with specific symptomatic manifestations. It is vital to bridge the gap between measurable patient characteristics, phenotypes, and underlying pathophysiological traits to enhance our understanding of OSA and its interplay with related outcomes. This knowledge could stimulate the development of tailored therapies targeting specific phenotypic and pathophysiological endotypes. This review aims to elucidate the multifaceted pathophysiology of OSA, focusing on the relationships between UA anatomy, functional traits, clinical manifestations, and comorbidities. The ultimate objective is to pave the way for a more personalized treatment paradigm in OSA, offering alternatives to continuous positive airway pressure therapy for selected patients and thereby optimizing treatment efficacy and adherence. There is an urgent need for personalized treatment strategies in the ever-evolving field of sleep medicine, as we progress from a ‘one-size-fits-all’ to a ‘tailored-therapy’ approach.

## 1. Introduction

Obstructive sleep apnea (OSA) represents a significant global health burden affecting an estimated 936 million adults globally with far-reaching consequences on individual and public health (1). OSA develops as a result of recurring upper airway obstruction (UA) during sleep leading to severely reduced or absent airflow (hypopnea or apnea). The disorder is typically associated with snoring and intermittent hypoxia, and episodes are frequently terminated by brief mini-arousals resulting in fragmented sleep with reduced amounts of slow wave sleep (SWS) and rapid-eye-movement (REM) sleep (2). These changes in sleep architecture may result in unrefreshing sleep and excessive daytime sleepiness (EDS) (3).

Beyond the direct impacts on sleep quality, OSA is associated with numerous comorbidities. The intermittent hypoxemia and sleep fragmentation associated with OSA can trigger cellular and molecular responses that promote sympathetic excitation, systemic inflammation, and other abnormal responses, which may result in the development of comorbidities such as cardiometabolic and neuropsychiatric conditions (4). OSA is also a major risk factor for motor vehicle accidents, which appears to be largely a consequence of EDS (5). There is strong evidence that untreated OSA is associated with systemic hypertension, especially with a loss of the normal nocturnal dipping pattern of blood pressure (BP), with growing evidence of risk for cardiovascular disease (6). Overall, these factors also lead to a substantial economic toll arising from direct medical expenditures, productivity losses, and accident-related costs (7). Despite its high prevalence and significant health implications, OSA remains underdiagnosed and undertreated, emphasizing the urgency for effective management strategies.

However, not all patients with OSA as measured by the level of sleep-disordered breathing (SDB), have a clinically significant disorder. Moreover, there is increasing evidence that the current grading of OSA severity as measured by the apnea-hypopnea index (AHI, number of apneas and hypopneas per hour of sleep) is inadequate (8). Additional measures including the hypoxic burden during sleep, the level of daytime symptoms such as sleepiness, and relevant biomarkers such as nocturnal BP dipping are required to adequately assess the clinical significance of the disorder in terms of outcomes, comorbidity risk, and treatment indications (9–12).

The standard therapy of OSA over the past 3 to 4 decades has centered around continuous positive airway pressure (CPAP), which acts by overcoming the negative intrapharyngeal pressure during inspiration that is the most important causative factor in OSA. While highly effective, the device is cumbersome, and compliance is limited. Thus, other treatment options are highly desirable, which can be facilitated by a detailed understanding of the complex pathophysiology of OSA. Since aspects of pathophysiology vary between patients, in addition to phenotypes, such an understanding should facilitate a personalized approach to management, especially in the area of pharmacotherapy. This consideration represents the principal objective of this review.

This review provides an overview of the pathophysiological and phenotypic factors of OSA in the context of therapeutic interventions, discusses their effectiveness in targeting different pathophysiological traits, and underscores the need for a shift toward personalized treatment modalities for optimal patient outcomes. Moreover, with the rise of new computational paradigms and machine learning approaches to categorizing and clustering patient symptom profiles, the review provides an overview of the topic and stresses the significance of connecting these novel techniques with understandable and quantifiable physiological factors to facilitate personalized treatments. To this end, we conducted a non-systematic literature search on PubMed and included studies and articles published up until July 2023. Our selection of articles was primarily guided by their relevance to the themes of pathophysiology, personalized treatment, and digital medicine techniques for OSA assessment and management. We sought to integrate the insights from these diverse sources into a narrative discussion, enriching the understanding of the current state of translating physiological factors and phenotypes into personalized treatment in its various aspects. This review does not aim to be exhaustive but aims to illuminate key concepts and stimulate further investigation in the realm of OSA pathophysiology, phenotypes, and personalized treatment.

## 2. Pathophysiology

**Figure 1:**
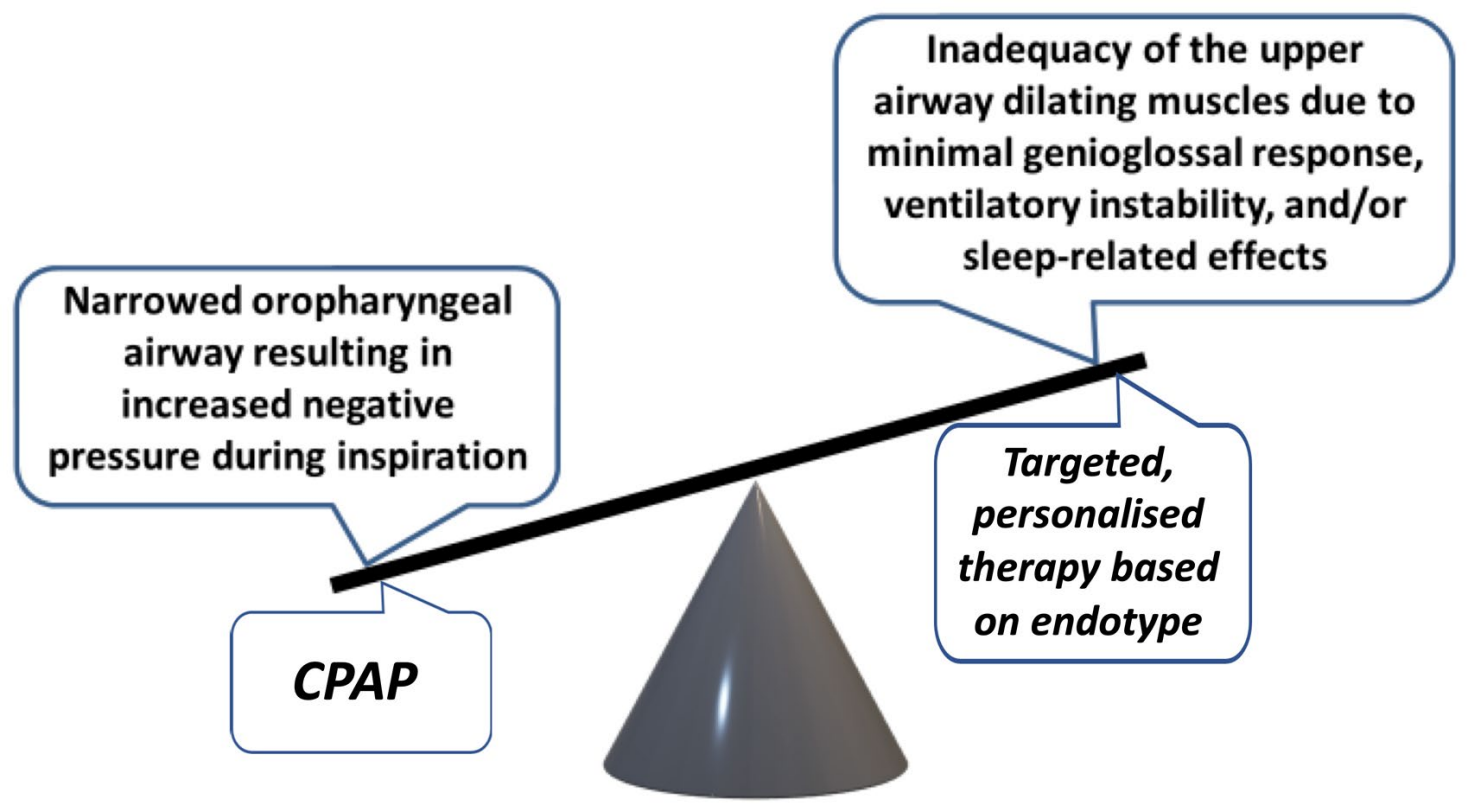
Balance of forces affecting the patency of the upper airway and potential for personalized treatment.

The pathophysiology of OSA is complex and multifactorial and stems from the interplay between anatomical and non-anatomical factors. The fundamental abnormality reflects an inability of the upper airway (UA) dilating muscles to overcome the negative forces that develop within the oropharynx during inspiration (Figure 1). The UA dilating muscles, contracting in a phasic manner that precede each inspiration, work to counteract the negative pressure generated in the UA during inspiration. This delicate balance can be compromised by any factor that escalates this negative pressure or diminishes the effectiveness of UA dilating muscle contractions, thereby leading to an increased risk of UA obstruction (2). A similar risk arises from a narrowed UA, as this amplifies the negative pressure in the oropharynx during inhalation, predisposing to closure. Anatomical factors that may contribute to such narrowing include craniofacial bony morphology, soft tissue accumulation in the neck from obesity or adenotonsillar hypertrophy, and variable factors such as fluid gravitating to the neck in the recumbent position. Moreover, non-anatomical factors such as diminished muscle responsiveness, heightened sensitivity to arousals, and a high ventilatory control system gain (termed loop gain), contribute significantly to the disorder’s development and progression, which are also influenced by genetic, environmental, and lifestyle factors.

### 2.1. Upper airway narrowing

The majority of patients with OSA have a narrowed oropharyngeal airway, a finding that can be clinically assessed by the Mallampati score (13). Genetic factors play a major role in this narrowing (14). Defects in the bony and maxillofacial structures in the lower face and neck as well as malocclusions, i.e., misalignments of the teeth when the jaws are closed, significantly contribute to UA narrowing (15). Maxillofacial defects can include conditions such as micrognathia, where the lower jaw is undersized, or retrognathia, characterized by a receding jawline. Both these conditions can result in posterior positioning of the tongue, consequently predisposing to obstruction of the UA (16). These defects may be evident in children with the Robin sequence or Treacher-Collins syndrome who are especially prone to OSA because of bony changes to the lower face and/or mandible (17). Similarly, a high-arched palate or a long soft palate can further reduce the size of the UA, thus contributing to OSA. Moreover, malocclusions often present with a retruded mandible, which may cause posterior positioning of the tongue. Alternatively, malocclusions may lead to difficulties in breathing through the nose causing increased mouth breathing and increasing the likelihood of OSA (18, 19).

Furthermore, the accumulation of soft tissue in the neck due to obesity or within the UA due to adenotonsillar hypertrophy can precipitate OSA in susceptible individuals by reducing the size of the oropharyngeal lumen (2). Conditions such as heart failure and end-stage renal failure associated with fluid retention can also contribute to the development of UA obstruction and OSA. This is due to nocturnal redistribution of fluid to the neck region while in a recumbent position, subsequently increasing UA collapsibility by narrowing of the lumen and diminishing the efficiency of dilator muscle contractions (20, 21).

While nasal obstruction is a relatively minor factor in predisposing to UA obstruction, variable nasal obstruction such as with rhinitis, contributes to the pathophysiology of OSA (22, 23). Furthermore, intranasal corticosteroid therapy has been reported to benefit patients with rhinitis and mild to moderate OSA (24). The supine body position may also compromise UA patency (25), largely due to gravitational forces, which is evident in the clinical setting where the AHI is often highest in the supine position.

### 2.2. Upper airway dilator muscle function

Patency of the UA is dependent on contraction of the pharyngeal dilator muscles, especially the genioglossus, which stiffen the collapsible segment of the UA during inspiration (2). The phasic contraction of these muscles is co-ordinated with inspiration and precede contraction of the diaphragm by milliseconds (26). Contraction of these UA muscles is influenced by chemical stimuli, vagal input, changes in UA pressure, and baroreceptor activity (27).

The narrowed UA seen in OSA results in greater inspiratory negative pressure, which requires more forceful contraction of the UA dilating muscles to maintain oropharyngeal patency. There is evidence that dilating muscle contraction in OSA is greater than in normal subjects during wakefulness, but diminishes to a greater extent during sleep, which predisposes to occlusion (28), especially in REM sleep (29). Hence, the primary issue in OSA is inadequate muscle compensation to combat the heightened inspiratory negative pressure, and not necessarily a fundamental deficiency in muscle function. The effects of this insufficient response by UA dilating muscles are aggravated by the fact that these are skeletal muscles, whose performance sees a more pronounced drop during sleep compared to the diaphragm.

### 2.3. Respiratory control

Respiratory control is an integral aspect of OSA pathophysiology and its dysfunction contributes to decreased UA muscle activity in certain circumstances. OSA often presents with a pattern of frequently reoccurring apneas, highlighting the instability of respiratory control which shares similarities to periodic breathing. At the heart of this control is the coordinated activity of the key muscles, diaphragm and genioglossus as UA obstruction is most likely to develop when EMG activity of these muscles is at the lowest point of the respiratory cycle, thus acting as a critical physiological determinant of apnea onset (2). As the apnea progresses, EMG activity of the UA dilating muscles progressively increases reaching a peak at apnea termination. This is typically followed by several large breaths after which both EMGs decrease potentially predisposing to further obstruction (30).

The transition from wakefulness to non-REM sleep usually results in a minor reduction in ventilation even in healthy subjects. This is attributed to a reduced response to the carbon dioxide (CO2) stimulus that drives respiration. However, in OSA patients, this decrease may tip the balance toward an apneic threshold that is critically dependent on CO2 levels (31). This is exacerbated by post-apnea hyperventilation, resulting in CO2 reduction and predisposing to further apneas (30).

A vital component of unstable respiratory control is loop gain, which is a measure of the sensitivity of the feedback loop that modifies ventilation in response to respiratory disturbances. As such, loop gain may also affect the predisposition to apnea. A high loop gain, where the magnitude of the increase in ventilation following apnea is high, contributes to ventilatory instability, thereby predisposing to recurring apnea (30).

Apnea termination is often associated with cortical arousal, which is an important protective mechanism, but may predispose to further apnea by contributing to post-apneic hyperventilation (32–34). These respiratory-related cortical arousals may vary in intensity and represent a distinct pathophysiologic feature (35), which may be quantified by the arousal threshold, that can be assessed noninvasively by PSG (36). A low arousal threshold may be a contributing factor to recurring apneas and represents a potential therapeutic target in selected patients (37). The interactions of these multiple elements underline the central role of respiratory control in the pathophysiology of OSA.

### 2.4. Genetic contribution

Emerging research highlights the significant role of genetics in the pathophysiology of OSA. Studies have demonstrated a substantial genetic component to OSA and related traits, such as BMI, craniofacial structure, and sleep-related parameters. There is a strong heritability in the size of the oropharyngeal space, which is a major factor in the UA narrowing that is typical of patients with OSA (14). Recent advances in genomics have further enabled the identification of specific genetic variants associated with OSA (38, 39). Polymorphisms in several genes, including those involved in serotonin metabolism, inflammation, and obesity, have been linked to OSA susceptibility (38). Furthermore, gene–environment interactions, particularly with obesity, also play a crucial role in OSA risk. These findings underscore the complex, multifactorial nature of OSA, with genetic factors interplaying with environmental and lifestyle factors in shaping disease onset and progression. While these advancements provide insights into the pathogenesis of OSA, there is still much to uncover, and ongoing research in this area is vital for refining disease risk prediction and uncovering potential targets for personalized treatment strategies.

### 2.5. Integrated pathophysiology and implications for treatment

Recently, there has been a surge in interest concerning the influence of non-anatomical factors on the development and progression of OSA (40). A study of subjects with and without OSA revealed that about one-third of the subjects in each group demonstrated the endotypes of either diminished genioglossus muscle responsiveness during sleep, low arousal threshold, or high loop gain, while 28% of subjects demonstrated a combination of multiple traits (41).

While the principal factor of increased UA collapsibility in OSA can be effectively reversed by CPAP therapy, a more comprehensive insight into the underlying pathophysiology provides the potential for additional management options in selected patients (Figure 1) (42). Inadequate UA dilating muscle compensation against increased collapsing forces may be improved by drug therapy that stimulates these muscles (43). The reduction in respiratory motor neurone output that is a physiological feature of sleep may be reversed by electrical stimulation of the hypoglossal nerve (44). A high loop gain may be diminished by acetazolamide (45) and a low arousal threshold may be increased by zolpidem therapy (46). These medications may have a role in selected patients where such factors are found to be contributing factors in the integrated pathophysiology of the disorder.

## 3. Clinical and pathophysiological phenotypes

### 3.1. Clinical phenotypes

The categorization of distinct clinical phenotypes is viable in populations suspected of having OSA (47). Furthermore, some pathophysiological traits that are frequently observed in OSA patients, such as loss of nocturnal dipping of BP, may influence the likelihood of associated comorbidity (48). As such, the findings of a diagnostic sleep study should be interpreted alongside these additional elements when assessing the clinical relevance of OSA for each patient. It’s essential to tailor management strategies to individual phenotypes, considering not only the symptom profile but additional factors beyond the AHI such as acute systemic effects and associated relevant comorbidities in the decision-making process (40). However, understanding the underlying pathophysiological mechanisms and connecting them to observable characteristics is crucial when moving toward individualized treatment pathways.

Endotypes and phenotypes of OSA have been extensively studied (49). Phenotype refers to a combination of disease characteristics that can be used to distinguish certain categories of patients from others (50) and several cluster analyses have been reported that distinguish clinical subtypes (51). Early reports identified 3 symptomatic phenotypes of OSA, namely disturbed sleep, minimal symptoms, and EDS (52). More recent reports added the additional phenotype where upper airway symptoms were dominant (53). An important feature of such reports is that similar average AHI levels were evident across clusters, which indicates that clusters of clinical phenotypes cannot be differentiated by the AHI. Furthermore, specific pathophysiological endotypes identified by PSG predicted the risk of adverse cardiovascular outcomes (54).

There is some evidence that the sleepy OSA phenotype may be associated with a higher risk of comorbidity (40), although this relationship is not clear-cut. A report based on the Sleep Heart Health Study indicated that the excessively sleepy phenotype was strongly associated with prevalent heart failure and incident cardiovascular disease (55). However, the insomnia subtype, which was a distinct cluster in a report from the European Sleep Apnoea Database cohort study (ESADA), was more frequently linked with cardiovascular comorbidity than the sleepy phenotype (56). The phenotype of non-dipping nocturnal BP has a high diagnostic prediction for OSA as measured by the AHI (57).

Future research requires the identification of specific markers of OSA that predict clinical significance and risk of adverse outcomes, and which may more reliably predict response to targeted treatment (49, 58). Identifying the pathophysiological and endotypic connections would further the understanding of the underlying phenomenon to deepen the insight into the mechanisms through which the markers connect to outcomes.

### 3.2. Modern data analytical methods for phenotyping and the translation to treatment

Traditionally, the identified phenotypes are dependent on categorized or simplistic variables and metrics only considering a few aspects of the disorders. For example, by quantifying sleep disruption by the number of awakenings or the overall sleep architecture while connecting that to categories of questionnaire-quantified sleepiness (51, 59). However, there exists inherent variation in the quantified parameters and differences in their reporting. For example, there is always at least minor inter-scorer variability both in respiratory event scoring and sleep staging and major differences in scoring arousals from sleep (60). This variability is inherently propagated to all consequent analyses and assessments and may affect the identified phenotypes. Similarly, quantifying sleepiness based on questionnaires such as ESS is variable, especially noticeable between genders (61), and there can also be intra-individual differences depending on the timing of the questionnaire (62).

There are different ways to go beyond the current clinical practices in identifying disease characteristics. For example, in the sleep architecture, various methods to quantify sleep fragmentation and sleep microstructure have been presented (63–66). Meanwhile, there are possibilities to characterize respiratory events as well as nocturnal hypoxemia in more detail (10, 11, 67). Similarly, aside from only assessing sleepiness based on questionnaires, there are various objective measures as well as different tests to assess neurocognitive function and impairment which may provide more reliable outcome metrics. The major hindrance in adaptation is the massive workload required to obtain a sufficient dataset for identifying phenotypes based on microstructures.

The rise of machine learning and artificial intelligence alongside increased computational capacities has given rise to different ways to utilize the entirety of the collected data without limiting it to a few simplified metrics (67). As an example, these developments form a major objective of Sleep Revolution project, funded by the European Union Horizon 2020 Research and Innovation Programme (Grant no. 965417), which seeks to transform current diagnostic methods for sleep-disordered breathing by using machine learning tools to facilitate automatic scoring outside traditional boundaries (68). By using these tools together with phenotyping, the project seeks to identify novel variables and analysis techniques that may more accurately identify patients with a clinically significant OSA syndrome, thus facilitating more personalized patient management (69). Similarly, as with biomarkers, there have been advances utilizing the patient characteristics and sleep recording data to develop markers, which can be termed data-markers, connected to disease characteristics, severity and risk of comorbidities (70). As an example, there have been promising approaches in identifying subgroups or clusters of patients based on observable characteristics. One way of summarizing the findings is a division into common themes as formulated by Zinchuk and Yaggi (51): (1) Subtype A, consisting of younger, obese males with severe OSA and classic symptoms, responding well to CPAP treatment; (2) Subtype B, which includes older, obese males with severe OSA, frequent comorbidities, and minimal symptoms, responding less effectively to CPAP; (3) Subtype C, featuring middle-aged, mildly obese females suffering from insomnia and moderate to severe OSA, with mixed responses to CPAP; and (4) Subtype D, which encompasses younger, nonobese males with severe OSA and primary upper airway symptoms, displaying the lowest hypoxemia and comorbidity rates and limited CPAP success. Overall, these approaches provide new pathways to targeted treatments.

However, while there have been promising approaches, these warrant further research and require major amounts of data, likely possible to obtain only through multi-institutional collaborative efforts. The major limiting factor in the adaptation of novel data-markers and data-analytical metrics to the clinics is the lack of reliability, generalizability, and especially the transparency of the results. If the obtained data-marker or phenotype cannot be rigorously connected to pathophysiological and endotypic aspects, it is unlikely to gain widespread use and reliability in clinical practice. While the modern data-analytical approaches may provide clearer connections between symptomology and measurable characteristics and even provide novel therapeutical targets, connecting these to the underlying factors and physiological effects giving rise to the observable characteristics would further promote the adaptation to clinics and therapeutics.

## 4. Personalized treatment

### 4.1. Continuous positive airway pressure

The fundamental goal of treatment in OSA is to maintain UA patency and thereby stabilize breathing pattern and ensure adequate ventilation. Continuous positive airway pressure (CPAP) is the gold standard in OSA treatment, consistently demonstrating high efficacy in mitigating the disorder’s principal signs and symptoms when used appropriately. Its mode of action is delivering a positive air pressure via a mask to the UA, which effectively prevents airway collapse during sleep.

In the context of personalized treatment, CPAP’s efficacy can occasionally extend beyond its primary anatomical target of airway obstruction. The therapy proves effective in treating non-anatomical or non-traditional traits involved in OSA pathophysiology, such as individuals with a high arousal threshold or high loop gain. Modern CPAP devices employ intricate algorithms to adjust pressure according to the user’s individual requirements, thus facilitating more personalized treatment strategies.

### 4.2. Dental appliances

Although CPAP is highly effective in maintaining UA patency, the device is cumbersome and is often not well tolerated. Thus, alternative effective therapies are desirable, especially in patients with poor CPAP compliance. For example, mandibular advancement devices (MAD) are designed to push the lower jaw forward during sleep and these custom-fitted dental appliances enhance the UA size and reduce its propensity to collapse. MADs are especially suited to treat primary snoring and mild OSA (71), but are also reasonable alternatives in patients with more severe OSA who fail to tolerate CPAP.

In personalized OSA therapy, MADs primarily target anatomical contributors but can indirectly influence some non-anatomical traits as well. Their ability to adjust the level of mandibular advancement allows for patient comfort and therapeutic efficacy (72), enhancing treatment adherence and outcomes. MADs are also effective for treating bruxism alongside OSA (73, 74). They offer a promising step toward individualizing OSA treatment, with ongoing research set to further enhance their utility in this area. While reports comparing CPAP and MAD therapy found CPAP to be generally more effective in reducing AHI and EDS, the effects were similar in patients with milder OSA (75).

### 4.3. Pharmacotherapy

Several pharmacological agents have been evaluated that target different pathophysiological endotypes of OSA. While many such agents have been reported to benefit OSA in the form of reduced AHI, none are yet licensed to treat the disorder (76). Drug therapies that target pathophysiological traits such as UA collapsibility by increasing dilator muscle contraction, respiratory control abnormalities such as high loop gain, and low arousal threshold have each been identified to benefit OSA in selected patient populations.

Desipramine, a central nervous system norepinephrine reuptake inhibitor and a member of the tricyclic antidepressant (TCA) family, lessens the sleep-induced reduction of genioglossus activity and enhances pharyngeal stability in healthy individuals (77). It has been observed to lower the AHI in patients with OSA who exhibit insufficient genioglossus muscle adaptation (78). Another drug, atomoxetine, which also inhibits norepinephrine reuptake, in combination with an antimuscarinic agent, oxybutynin, has been found to considerably decrease the AHI in patients suffering from OSA (43).

Hypnotics have long been recommended to avoid in patients with respiratory disease because of potential adverse effects on respiration during sleep. However, recent reports in the setting of OSA indicate a potential role for certain hypnotics such as zolpidem in selected patients where a low arousal threshold represents a significant pathophysiological trait (37, 79). However, as there are no published randomized clinical trials, no recommendations can be given on the clinical use of hypnotics in patients with OSA (80). Another more recent report indicated that zolpidem increases sleep efficiency and the respiratory arousal threshold without changing sleep apnoea severity and pharyngeal muscle activity (46).

### 4.4. Surgical approaches

Accumulation of soft tissue in the oropharyngeal region contributing to airway constriction can be medically or surgically addressed. Pediatric patients showing signs of adenotonsillar hypertrophy and OSA can improve with surgical intervention (81), while adults with central obesity and OSA can benefit from weight loss through methods such as bariatric surgery (82) or intensive dietary control coupled with medication (83). Liraglutide, a long-acting agonist of the glucagon-like peptide one receptor, has shown promising results in inducing weight loss and significantly decreasing AHI in OSA patients (84).

### 4.5. Other approaches and digital medicine

The diminished output of respiratory motor neurons triggered by sleep could potentially be countered by electrical stimulation of the hypoglossal nerve, offering an alternative treatment to CPAP, particularly for patients who struggle with compliance (44, 85). For those with high loop gain, acetazolamide may provide relief for OSA symptoms and carry the added advantage of decreasing blood pressure (45, 86). For individuals suffering from OSA who also have fluid overload, diuretic therapy might be beneficial by curbing the nocturnal upward shift of fluid (87).

The role of oxygen therapy in the management of OSA is uncertain and not advised in most cases, although a recent report suggests that oxygen supplementation may benefit OSA acutely, possibly by reducing the arousal response (88). Finally, positional treatments in cases where most of the respiratory events occur in supine position have long been recognized as a viable alternative (89).

Advances in digital medicine have begun to revolutionize the prevention and treatment of OSA. Telemedicine and remote patient monitoring, for instance, are making it easier for healthcare providers to diagnose and manage OSA. Patients can undertake sleep studies in the comfort of their homes using portable polysomnography devices, while apps and wearable technologies are providing insights into sleep pattern and other critical factors related to OSA (68). Moreover, cognitive behavioral therapy for insomnia (CBT-I) is showing promise in managing comorbid conditions often present in OSA patients, and has potential for digitalization and simple utilization alongside other treatment modalities (90, 91). Additionally, machine learning and artificial intelligence algorithms have the potential in the future to be utilized to understand factors behind limited adherence for CPAP and MAD devices as well as to optimize their settings, thus enabling a more personalized approach to OSA treatment. These digital health tools not only enhance the accessibility and convenience of OSA treatment but also offer the potential for more effective, tailored therapeutic interventions.

**Figure 2:**
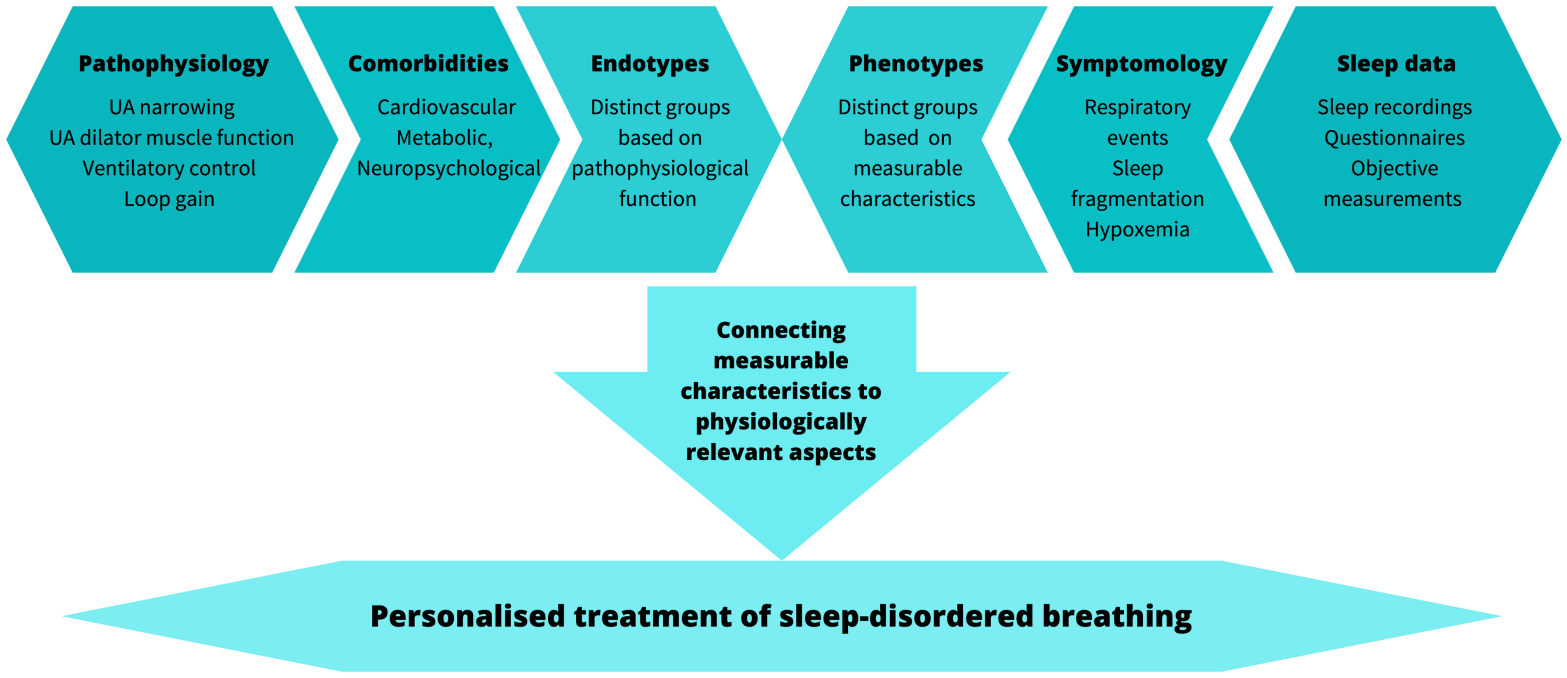
Factors to consider when translating pathophysiology and phenotypes to personalized treatment.

Overall, several potential treatment pathways have been identified (Table 1). However, targeting these to patients and choosing the optimal treatment pathway may require extensive knowledge and assessment of the pathophysiological characteristics and the individualized phenotype to match the established efficacy of CPAP (Figure 2).

**Table 1:** Summary of the key findings and implications for personalized treatment of obstructive sleep apnea (OSA).

Key findings	Implications for personalized treatment
OSA is characterized by recurrent upper airway obstruction during sleep due to an imbalance between negative inspiratory pressure and the ability of the upper airway dilating muscles to maintain patency	Continuous Positive Airway Pressure (CPAP) and Mandibular Advancement Devices (MADs) can be tailored to individual needs, but suffer from low compliance
Identifying individual pathophysiological traits can inform personalized treatment approaches	Personalized treatment strategies have the potential to provide alternatives to CPAP therapy in selected patients
OSA is often associated with comorbidities such as cardiovascular disease, metabolic disorders, and neuropsychological conditions	Novel treatment strategies such as hypoglossal nerve stimulation, certain medications, and lifestyle modifications like weight loss can be employed based on the patient’s unique pathophysiology
There are various phenotypes independent of the number of obstructions, which relate to symptoms and outcomes; however, linking these to underlying pathophysiology is vital to advancing our understanding of OSA	Ongoing research and advances in digital medicine techniques can further enhance the personalization of OSA treatment

## 5. Take-away messages and practical care points

Overall, successful management of OSA requires a comprehensive, personalized, and interdisciplinary approach. Below, we provide a few practical care points derived from our review, designed to assist in the decision-making process and enhance the management of OSA patients in a practical and applied manner:

1. **Comprehensive Evaluation**: OSA is multifactorial. A thorough evaluation of factors such as craniofacial anatomy, body mass index, neck circumference, and sleeping positions can provide valuable insights into a patient’s risk of OSA.
2. **Recognition of Comorbidities**: Given the significant association between OSA and various cardiovascular, metabolic, and neuropsychiatric conditions, screening for these comorbidities should be an integral part of the patient’s clinical evaluation.
3. **Phenotypic Consideration**: Understanding that OSA presents with different phenotypes can aid in the identification of patients who might not respond to conventional therapies. Awareness of these phenotypes can also help clinicians provide personalized treatment strategies.
4. **Alternative Treatments**: In patients who are non-compliant or non-responsive to CPAP therapy, consider other treatments such as dental devices, positional therapy, upper airway surgery, hypoglossal nerve stimulation, or pharmacotherapy.
5. **Proactive Follow-up and Management of OSA**: Regular follow-ups are necessary to assess the efficacy of the chosen treatment strategy and make adjustments if necessary. Also, patient education regarding the potential implications of untreated OSA and the benefits of treatment compliance can aid in improving therapeutic outcomes.
6. **Interdisciplinary Approach**: Clinicians should be encouraged to collaborate with experts from various disciplines, such as dietitians for weight management, psychologists for behavior therapy, or surgeons for potential surgical interventions, for a holistic approach to patient care.
7. **Research and Continuous Learning**: As the understanding of OSA pathophysiology and treatment options evolves, clinicians should make efforts to stay updated on the latest research findings and integrate them into their practice where relevant.

## 6. Conclusion and future directions

Despite extensive knowledge of the pathophysiological mechanisms and the adverse systemic effects of OSA, only limited benefit, if any, has been demonstrated from CPAP therapy in randomized trials designed to evaluate cardiometabolic benefit in patients with OSA. Many reasons can be considered for these negative outcomes including inappropriate patient selection such as inclusion of mainly non-sleepy patients, reliance on the AHI as the sole measure of disease categorization, inclusion of patients with pre-existing comorbidity, and inadequate CPAP compliance. Patient selection for such outcome studies should consider inclusion variables beyond the AHI that include symptomatic patients, and CPAP compliance should be factored into the outcome assessment (92).

Furthermore, it should be considered that some symptoms consistent with OSA such as sleepiness and fatigue could be a result of other factors such as lifestyle and disturbed sleep. New approaches to syndrome definition are required that consider different clinical OSA phenotypes in combination with endotypes and pathophysiological factors. New diagnostic approaches are needed that incorporate novel technologies to provide surrogates for sleep structure, to gauge exposure to systemic effects of OSA, and to identify specific biomarkers and data-markers for disease classification. While potentially useful markers could conceivably be derived from the PSG (93–96), the conventional sleep diagnostic test will likely require adaptation to facilitate ambulatory and multi-night diagnostic studies.

The ultimate goal is the development of diagnostic approaches that lead to the diagnosis of a clinically relevant OSA disorder that will include measures that give a more comprehensive insight into pathophysiological mechanisms in the individual patient. This approach should facilitate a more personalized treatment plan that goes beyond the simple question of CPAP or not (58, 97, 98).

## Author contributions

WM wrote the first draft of the manuscript and designed the concept and structure of the review. HK wrote sections of the manuscript and contributed to the conception of the review. All authors contributed to the manuscript revision, read, and approved the submitted version.

## Funding

This project was supported by funding from the European Union’s Horizon 2020 research and innovation programme under grant agreement no. 965417 and the Research Committee of the Kuopio University Hospital Catchment Area for the State Research Funding (project 5041803).

## Conflict of interest

The authors declare that the research was conducted in the absence of any commercial or financial relationships that could be construed as a potential conflict of interest.

## Publisher’s note

All claims expressed in this article are solely those of the authors and do not necessarily represent those of their affiliated organizations, or those of the publisher, the editors and the reviewers. Any product that may be evaluated in this article, or claim that may be made by its manufacturer, is not guaranteed or endorsed by the publisher.

## References

[1] Benjafield AV Ayas NT Eastwood PR Heinzer R Ip MSM Morrell MJ. Estimation of the global prevalence and burden of obstructive sleep apnoea: a literature-based analysis. Lancet Respir Med. (2019) 7:687–98. doi: 10.1016/S2213-2600(19)30198-5, PMID: 31300334 PMC7007763

[2] Deegan PC McNicholas WT. Pathophysiology of obstructive sleep apnoea. Eur Respir J. (1995) 8:1161–78. doi: 10.1183/09031936.95.08071161 7589402

[3] Levy P Kohler M McNicholas WT Barbe F McEvoy RD Somers VK. Obstructive sleep apnoea syndrome. Nat Rev Dis Primers. (2015) 1:15015. doi: 10.1038/nrdp.2015.15 27188535

[4] McNicholas WT. Obstructive sleep apnoea and comorbidity - an overview of the association and impact of continuous positive airway pressure therapy. Expert Rev Respir Med. (2019) 13:251–61. doi: 10.1080/17476348.2019.1575204, PMID: 30691323

[5] Bonsignore MR Marrone O Fanfulla F. Sleep apnea, sleepiness, and driving risk. Sleep Med Clin. (2019) 14:431–9. doi: 10.1016/j.jsmc.2019.08.001 31640871

[6] McNicholas WT Bonsigore MR Management Committee of ECAB. Sleep apnoea as an independent risk factor for cardiovascular disease: current evidence, basic mechanisms and research priorities. Eur Respir J. (2007) 29:156–78. doi: 10.1183/09031936.00027406, PMID: 17197482

[7] Lyons MM Bhatt NY Pack AI Magalang UJ. Global burden of sleep-disordered breathing and its implications. Respirology. (2020) 25:690–702. doi: 10.1111/resp.13838, PMID: 32436658

[8] Pevernagie DA Gnidovec-Strazisar B Grote L Heinzer R McNicholas WT Penzel T. On the rise and fall of the apnea-hypopnea index: a historical review and critical appraisal. J Sleep Res. (2020) 29:e13066. doi: 10.1111/jsr.13066, PMID: 32406974

[9] McNicholas WT Pevernagie D. Obstructive sleep apnea: transition from pathophysiology to an integrative disease model. J Sleep Res. (2022) 31:e13616. doi: 10.1111/jsr.13616, PMID: 35609941 PMC9539471

[10] Pahari P Korkalainen H Karhu T Rissanen M Arnardottir ES Hrubos-Strøm H. Obstructive sleep apnea-related intermittent hypoxaemia is associated with impaired vigilance. J Sleep Res. (2023) 32:e13803. doi: 10.1111/jsr.13803, PMID: 36482788

[11] Kainulainen S Duce B Korkalainen H Oksenberg A Leino A Arnardottir ES. Severe desaturations increase psychomotor vigilance task-based median reaction time and number of lapses in obstructive sleep apnoea patients. Eur Respir J. (2020) 55:1901849. doi: 10.1183/13993003.01849-2019, PMID: 32029446 PMC7142879

[12] Kainulainen S Töyräs J Oksenberg A Korkalainen H Sefa S Kulkas A. Severity of desaturations reflects OSA-related daytime sleepiness better than AHI. J Clin Sleep Med. (2019) 15:1135–42. doi: 10.5664/jcsm.7806 31482835 PMC6707054

[13] Yu JL Rosen I. Utility of the modified Mallampati grade and Friedman tongue position in the assessment of obstructive sleep apnea. J Clin Sleep Med. (2020) 16:303–8. doi: 10.5664/jcsm.8188, PMID: 31992434 PMC7053038

[14] Chi L Comyn FL Keenan BT Cater J Maislin G Pack AI. Heritability of craniofacial structures in normal subjects and patients with sleep apnea. Sleep. (2014) 37:1689–98. doi: 10.5665/sleep.4082, PMID: 25197806 PMC4173925

[15] Neelapu BC Kharbanda OP Sardana HK Balachandran R Sardana V Kapoor P. Craniofacial and upper airway morphology in adult obstructive sleep apnea patients: a systematic review and meta-analysis of cephalometric studies. Sleep Med Rev. (2017) 31:79–90. doi: 10.1016/j.smrv.2016.01.007, PMID: 27039222

[16] McNicholas WT. Diagnosis of obstructive sleep apnea in adults. Proc Am Thorac Soc. (2008) 5:154–60. doi: 10.1513/pats.200708-118MG 18250207

[17] Tan HL Kheirandish-Gozal L Abel F Gozal D. Craniofacial syndromes and sleep-related breathing disorders. Sleep Med Rev. (2016) 27:74–88. doi: 10.1016/j.smrv.2015.05.010, PMID: 26454241 PMC5374513

[18] Dastan F Ghaffari H Shishvan HH Zareiyan M Akhlaghian M Shahab S. Correlation between the upper airway volume and the hyoid bone position, palatal depth, nasal septum deviation, and concha bullosa in different types of malocclusion: a retrospective cone-beam computed tomography study. Dental Med Problems. (2021) 58:509–14. doi: 10.17219/dmp/130099, PMID: 34850611

[19] Galeotti A Festa P Viarani V D'Antò V Sitzia E Piga S. Prevalence of malocclusion in children with obstructive sleep apnoea. Orthod Craniofac Res. (2018) 21:242–7. doi: 10.1111/ocr.12242, PMID: 30188002

[20] White LH Bradley TD. Role of nocturnal rostral fluid shift in the pathogenesis of obstructive and central sleep apnoea. J Physiol. (2013) 591:1179–93. doi: 10.1113/jphysiol.2012.245159, PMID: 23230237 PMC3607865

[21] Lyons OD Inami T Perger E Yadollahi A Chan CT Bradley TD. The effect of fluid overload on sleep apnoea severity in haemodialysis patients. Eur Respir J. (2017) 49:1601789. doi: 10.1183/13993003.01789-2016, PMID: 28381432

[22] McNicholas WT. The nose and OSA: variable nasal obstruction may be more important in pathophysiology than fixed obstruction. Eur Res J. (2008) 32:3–8. doi: 10.1183/09031936.00050208, PMID: 18591332

[23] McNicholas WT Tarlo S Cole P Zamel N Rutherford R Griffin D. Obstructive apneas during sleep in patients with seasonal allergic rhinitis. Am Rev Respir Dis. (1982) 126:625–8. PMID: 7125355 10.1164/arrd.1982.126.4.625

[24] Kiely JL Nolan P McNicholas WT. Intranasal corticosteroid therapy for obstructive sleep apnoea in patients with co-existing rhinitis. Thorax. (2004) 59:50–5. PMID: 14694248 PMC1758841

[25] Yildirim N Fitzpatrick MF Whyte KF Jalleh R Wightman AJ Douglas NJ. The effect of posture on upper airway dimensions in normal subjects and in patients with the sleep apnea/hypopnea syndrome. Am Rev Respir Dis. (1991) 144:845–7. doi: 10.1164/ajrccm/144.4.845, PMID: 1928960

[26] Strohl KP Hensley MJ Hallett M Saunders NA Ingram RH Jr. Activation of upper airway muscles before onset of inspiration in normal humans. J Appl Physiol Respir Environ Exerc Physiol. (1980) 49:638–42. doi: 10.1152/jappl.1980.49.4.638 6777347

[27] Brouillette RT Thach BT. Control of genioglossus muscle inspiratory activity. J Appl Physiol Respir Environ Exerc Physiol. (1980) 49:801–8. doi: 10.1152/jappl.1980.49.5.801, PMID: 6776078

[28] Mezzanotte WS Tangel DJ White DP. Influence of sleep onset on upper-airway muscle activity in apnea patients versus normal controls. Am J Respir Crit Care Med. (1996) 153:1880–7. doi: 10.1164/ajrccm.153.6.8665050, PMID: 8665050

[29] Carberry JC Jordan AS White DP Wellman A Eckert DJ. Upper airway collapsibility (Pcrit) and pharyngeal dilator muscle activity are sleep stage dependent. Sleep. (2016) 39:511–21. doi: 10.5665/sleep.5516, PMID: 26612386 PMC4763361

[30] Dempsey JA Veasey SC Morgan BJ O'Donnell CP. Pathophysiology of sleep apnea. Physiol Rev. (2010) 90:47–112. doi: 10.1152/physrev.00043.2008, PMID: 20086074 PMC3970937

[31] Phillipson EA. Control of breathing during sleep. Am Rev Respir Dis. (1978) 118:909–39. PMID: 216294 10.1164/arrd.1978.118.5.909

[32] Eckert DJ Malhotra A. Pathophysiology of adult obstructive sleep apnea. Proc Am Thorac Soc. (2008) 5:144–53. doi: 10.1513/pats.200707-114MG, PMID: 18250206 PMC2628457

[33] McNicholas WT. Arousal in the sleep apnoea syndrome: a mixed blessing? Eur Resp J. (1998) 12:1239–41. doi: 10.1183/09031936.98.12061239, PMID: 9877469

[34] Jordan AS Wellman A Heinzer RC Lo YL Schory K Dover L. Mechanisms used to restore ventilation after partial upper airway collapse during sleep in humans. Thorax. (2007) 62:861–7. doi: 10.1136/thx.2006.070300, PMID: 17412778 PMC2094262

[35] Bahr K Geisler V Huppertz T Groppa S Matthias C Gouveris H. Intensity of respiratory cortical arousals is a distinct pathophysiologic feature and is associated with disease severity in obstructive sleep apnea patients. Brain Sci. (2021) 11:282. doi: 10.3390/brainsci11030282, PMID: 33668974 PMC7996607

[36] Sands SA Terrill PI Edwards BA Taranto Montemurro L Azarbarzin A Marques M. Quantifying the arousal threshold using polysomnography in obstructive sleep apnea. Sleep. (2018) 41:zsx183. doi: 10.1093/sleep/zsx183, PMID: 29228393 PMC5804982

[37] Eckert DJ Owens RL Kehlmann GB Wellman A Rahangdale S Yim-Yeh S. Eszopiclone increases the respiratory arousal threshold and lowers the apnoea/hypopnoea index in obstructive sleep apnoea patients with a low arousal threshold. Clin Sci. (2011) 120:505–14. doi: 10.1042/CS20100588 PMC3415379 21269278

[38] Xu H Liu F Li Z Li X Liu Y Li N. Genome-wide association study of obstructive sleep apnea and objective sleep-related traits identifies novel risk loci in Han Chinese individuals. Am J Respir Crit Care Med. (2022) 206:1534–45. doi: 10.1164/rccm.202109-2044OC 35819321

[39] Wieckiewicz M Bogunia-Kubik K Mazur G Danel D Smardz J Wojakowska A. Genetic basis of sleep bruxism and sleep apnea-response to a medical puzzle. Sci Rep. (2020) 10:7497. doi: 10.1038/s41598-020-64615-y, PMID: 32367059 PMC7198562

[40] Randerath W Bassetti CL Bonsignore MR Farre R Ferini-Strambi L Grote L. Challenges and perspectives in obstructive sleep apnoea: report by an ad hoc working group of the sleep disordered breathing Group of the European Respiratory Society and the European Sleep Research Society. Eur Respir J. (2018) 52:1702616. doi: 10.1183/13993003.02616-2017 29853491

[41] Eckert DJ White DP Jordan AS Malhotra A Wellman A. Defining phenotypic causes of obstructive sleep apnea. Identification of novel therapeutic targets. Am J Respir Crit Care Med. (2013) 188:996–1004. doi: 10.1164/rccm.201303-0448OC 23721582 PMC3826282

[42] Schutz SG Dunn A Braley TJ Pitt B Shelgikar AV. New frontiers in pharmacologic obstructive sleep apnea treatment: a narrative review. Sleep Med Rev. (2021) 57:101473. doi: 10.1016/j.smrv.2021.101473, PMID: 33853035

[43] Taranto-Montemurro L Messineo L Sands SA Azarbarzin A Marques M Edwards BA. The combination of atomoxetine and oxybutynin greatly reduces obstructive sleep apnea severity. A randomized, placebo-controlled, double-blind crossover trial. Am J Respir Crit Care Med. (2019) 199:1267–76. doi: 10.1164/rccm.201808-1493OC 30395486 PMC6519859

[44] Strollo PJ Jr Soose RJ Maurer JT de Vries N Cornelius J Froymovich O. Upper-airway stimulation for obstructive sleep apnea. N Engl J Med. (2014) 370:139–49. doi: 10.1056/NEJMoa1308659 24401051

[45] Edwards BA Sands SA Eckert DJ White DP Butler JP Owens RL. Acetazolamide improves loop gain but not the other physiological traits causing obstructive sleep apnoea. J Physiol. (2012) 590:1199–211. doi: 10.1113/jphysiol.2011.223925, PMID: 22219335 PMC3381825

[46] Messineo L Eckert DJ Lim R Chiang A Azarbarzin A Carter SG. Zolpidem increases sleep efficiency and the respiratory arousal threshold without changing sleep apnoea severity and pharyngeal muscle activity. J Physiol. (2020) 598:4681–92. doi: 10.1113/JP280173, PMID: 32864734

[47] Bailly S Grote L Hedner J Schiza S McNicholas WT Basoglu OK. Clusters of sleep apnoea phenotypes: a large pan-European study from the European sleep apnoea database (ESADA). Respirology. (2020) 26:378–87. doi: 10.1111/resp.13969 33140467

[48] Crinion SJ Ryan S McNicholas WT. Obstructive sleep apnoea as a cause of nocturnal nondipping blood pressure: recent evidence regarding clinical importance and underlying mechanisms. Eur Respir J. (2017) 49:1601818. doi: 10.1183/13993003.01818-2016, PMID: 28077479

[49] Edwards BA Redline S Sands SA Owens RL. More than the sum of the respiratory events: personalized medicine approaches for obstructive sleep apnea. Am J Respir Crit Care Med. (2019) 200:691–703. doi: 10.1164/rccm.201901-0014TR, PMID: 31022356 PMC6775874

[50] Zinchuk AV Gentry MJ Concato J Yaggi HK. Phenotypes in obstructive sleep apnea: a definition, examples and evolution of approaches. Sleep Med Rev. (2017) 35:113–23. doi: 10.1016/j.smrv.2016.10.002, PMID: 27815038 PMC5389934

[51] Zinchuk AV Yaggi HK. Phenotypic subtypes of OSA: a challenge and opportunity for precision medicine. Chest. (2020) 157:403–20. doi: 10.1016/j.chest.2019.09.002, PMID: 31539538 PMC7005379

[52] Ye L Pien GW Ratcliffe SJ Bjornsdottir E Arnardottir ES Pack AI. The different clinical faces of obstructive sleep apnoea: a cluster analysis. Eur Respir J. (2014) 44:1600–7. doi: 10.1183/09031936.00032314, PMID: 25186268 PMC6675398

[53] Keenan BT Kim J Singh B Bittencourt L Chen NH Cistulli PA. Recognizable clinical subtypes of obstructive sleep apnea across international sleep centers: a cluster analysis. Sleep. (2018) 41:zsx214. doi: 10.1093/sleep/zsx214, PMID: 29315434 PMC5914381

[54] Zinchuk AV Yaggi HK. Sleep apnea heterogeneity, phenotypes, and cardiovascular risk. Implications for trial design and precision sleep medicine. Am J Respir Crit Care Med. (2019) 200:412–3. doi: 10.1164/rccm.201903-0545ED 30916985 PMC6701039

[55] Mazzotti DR Keenan BT Lim DC Gottlieb DJ Kim J Pack AI. Symptom subtypes of obstructive sleep apnea predict incidence of cardiovascular outcomes. Am J Respir Crit Care Med. (2019) 200:493–506. doi: 10.1164/rccm.201808-1509OC, PMID: 30764637 PMC6701040

[56] Saaresranta T Hedner J Bonsignore MR Riha RL McNicholas WT Penzel T. Clinical phenotypes and comorbidity in European sleep apnoea patients. PLoS One. (2016) 11:e0163439. doi: 10.1371/journal.pone.0163439, PMID: 27701416 PMC5049787

[57] Crinion SJ Ryan S Kleinerova J Kent BD Gallagher J Ledwidge M. Nondipping nocturnal blood pressure predicts sleep apnea in patients with hypertension. J Clin Sleep Med. (2019) 15:957–63. doi: 10.5664/jcsm.7870, PMID: 31383232 PMC6622521

[58] Pevernagie D. Future treatment of sleep disorders: syndromic approach versus management of treatable traits? Sleep Med Clin. (2021) 16:465–73. doi: 10.1016/j.jsmc.2021.05.005, PMID: 34325823

[59] Bianchi MT Russo K Gabbidon H Smith T Goparaju B Westover MB. Big data in sleep medicine: prospects and pitfalls in phenotyping. Nat Sci Sleep. (2017) 9:11–29. doi: 10.2147/NSS.S130141, PMID: 28243157 PMC5317347

[60] Magalang UJ Chen NH Cistulli PA Fedson AC Gíslason T Hillman D. Agreement in the scoring of respiratory events and sleep among international sleep centers. Sleep. (2013) 36:591–6. doi: 10.5665/sleep.2552, PMID: 23565005 PMC3612261

[61] Martínez-García M Labarca G. Obstructive sleep apnea in women: scientific evidence is urgently needed. J Clin Sleep Med. (2022) 18:1–2. doi: 10.5664/jcsm.9684, PMID: 34648424 PMC8807894

[62] Grewe FA Roeder M Bradicich M Schwarz EI Held U Thiel S. Low repeatability of Epworth sleepiness Scale after short intervals in a sleep clinic population. J Clin Sleep Med. (2020) 16:757–64. doi: 10.5664/jcsm.8350, PMID: 32039756 PMC7849809

[63] Terzano MG Mancia D Salati MR Costani G Decembrino A Parrino L. The cyclic alternating pattern as a physiologic component of normal NREM sleep. Sleep. (1985) 8:137–45. doi: 10.1093/sleep/8.2.137, PMID: 4012156

[64] Younes M Ostrowski M Soiferman M Younes H Younes M Raneri J. Odds ratio product of sleep EEG as a continuous measure of sleep state. Sleep. (2015) 38:641–54. doi: 10.5665/sleep.4588 25348125 PMC4355904

[65] Korkalainen H Leppanen T Duce B Kainulainen S Aakko J Leino A. Detailed assessment of sleep architecture with deep learning and shorter epoch-to-epoch duration reveals sleep fragmentation of patients with obstructive sleep apnea. IEEE J Biomed Health Inform. (2021) 25:2567–74. doi: 10.1109/JBHI.2020.3043507, PMID: 33296317

[66] Huttunen R Leppänen T Duce B Oksenberg A Myllymaa S Töyräs J. Assessment of obstructive sleep apnea-related sleep fragmentation utilizing deep learning-based sleep staging from photoplethysmography. Sleep. (2021) 44:zsab142. doi: 10.1093/sleep/zsab142, PMID: 34089616 PMC8503836

[67] Rissanen M Korkalainen H Duce B Sillanmaki S Pitkanen H Suni A. Obstructive sleep apnea patients with atrial arrhythmias suffer from prolonged recovery from desaturations. IEEE Trans Biomed Eng. (2023) 70:2122–30. doi: 10.1109/TBME.2023.3236680 37018722

[68] Arnardottir ES Islind AS Óskarsdóttir M Ólafsdóttir KA August E Jónasdóttir L. The sleep revolution project: the concept and objectives. J Sleep Res. (2022) 31:e13630. doi: 10.1111/jsr.13630, PMID: 35770626

[69] McNicholas WT Arnardottir ES Leppänen T Schiza S Randerath W. CPAP therapy for obstructive sleep apnoea: persisting challenges in outcome assessment. Eur Respir J. (2023) 62:2300182. doi: 10.1183/13993003.00182-2023, PMID: 37474148

[70] Finnsson E Arnardóttir E Cheng W-J Alex RM Sigmarsdóttir ÞB Helgason S. Sleep apnea endotypes: from the physiological laboratory to scalable polysomnographic measures. Front Sleep. (2023) 2:1188052. doi: 10.3389/frsle.2023.1188052 PMC12713878 41426478

[71] Ramar K Dort LC Katz SG Lettieri CJ Harrod CG Thomas SM. Clinical practice guideline for the treatment of obstructive sleep apnea and snoring with oral appliance therapy: an update for 2015. J Clin Sleep Med. (2015) 11:773–827. doi: 10.5664/jcsm.4858, PMID: 26094920 PMC4481062

[72] Shi X Lobbezoo F Chen H Rosenmöller B Berkhout E de Lange J. Comparisons of the effects of two types of titratable mandibular advancement devices on respiratory parameters and upper airway dimensions in patients with obstructive sleep apnea: a randomized controlled trial. Clin Oral Investig. (2023) 27:2013–25. doi: 10.1007/s00784-023-04945-z, PMID: 36928350 PMC10160211

[73] Martynowicz H Wieczorek T Macek P Wojakowska A Poręba R Gać P. The effect of continuous positive airway pressure and mandibular advancement device on sleep bruxism intensity in obstructive sleep apnea patients. Chron Respir Dis. (2022) 19:147997312110523. doi: 10.1177/14799731211052301 PMC9081718 35512250

[74] Wojda M Kostrzewa-Janicka J. Influence of MAD application on episodes of obstructive apnea and bruxism during sleep-a prospective study. J Clin Med. (2022) 11:5809. doi: 10.3390/jcm11195809, PMID: 36233677 PMC9570562

[75] Sharples LD Clutterbuck-James AL Glover MJ Bennett MS Chadwick R Pittman MA. Meta-analysis of randomised controlled trials of oral mandibular advancement devices and continuous positive airway pressure for obstructive sleep apnoea-hypopnoea. Sleep Med Rev. (2016) 27:108–24. doi: 10.1016/j.smrv.2015.05.003, PMID: 26163056 PMC5378304

[76] Gaisl T Haile SR Thiel S Osswald M Kohler M. Efficacy of pharmacotherapy for OSA in adults: a systematic review and network meta-analysis. Sleep Med Rev. (2019) 46:74–86. doi: 10.1016/j.smrv.2019.04.009, PMID: 31075665

[77] Taranto-Montemurro L Edwards BA Sands SA Marques M Eckert DJ White DP. Desipramine increases genioglossus activity and reduces upper airway collapsibility during non-REM sleep in healthy subjects. Am J Respir Crit Care Med. (2016) 194:878–85. doi: 10.1164/rccm.201511-2172OC, PMID: 26967681 PMC5074653

[78] Taranto-Montemurro L Sands SA Edwards BA Azarbarzin A Marques M de Melo C. Desipramine improves upper airway collapsibility and reduces OSA severity in patients with minimal muscle compensation. Eur Respir J. (2016) 48:1340–50. doi: 10.1183/13993003.00823-2016, PMID: 27799387 PMC5437721

[79] Carberry JC Fisher LP Grunstein RR Gandevia SC McKenzie DK Butler JE. Role of common hypnotics on the phenotypic causes of obstructive sleep apnoea: paradoxical effects of zolpidem. Eur Respir J. (2017) 50:1701344. doi: 10.1183/13993003.01344-2017, PMID: 29284686

[80] Carberry JC Amatoury J Eckert DJ. Personalized management approach for OSA. Chest. (2018) 153:744–55. doi: 10.1016/j.chest.2017.06.011 28629917

[81] Stradling JR Thomas G Warley AR Williams P Freeland A. Effect of adenotonsillectomy on nocturnal hypoxaemia, sleep disturbance, and symptoms in snoring children. Lancet. (1990) 335:249–53. doi: 10.1016/0140-6736(90)90068-G, PMID: 1967719

[82] Currie AC Kaur V Carey I Al-Rubaye H Mahawar K Madhok B. Obstructive sleep apnea remission following bariatric surgery: a national registry cohort study. Surg Obes Relat Dis. (2021) 17:1576–82. doi: 10.1016/j.soard.2021.05.021, PMID: 34187745

[83] Chirinos JA Gurubhagavatula I Teff K Rader DJ Wadden TA Townsend R. CPAP, weight loss, or both for obstructive sleep apnea. N Engl J Med. (2014) 370:2265–75. doi: 10.1056/NEJMoa1306187, PMID: 24918371 PMC4138510

[84] Blackman A Foster GD Zammit G Rosenberg R Aronne L Wadden T. Effect of liraglutide 3.0 mg in individuals with obesity and moderate or severe obstructive sleep apnea: the SCALE sleep apnea randomized clinical trial. Int J Obes. (2016) 40:1310–9. doi: 10.1038/ijo.2016.52, PMID: 27005405 PMC4973216

[85] Heiser C Steffen A Hofauer B Mehra R Strollo PJ Jr Vanderveken OM. Effect of upper airway stimulation in patients with obstructive sleep apnea (EFFECT): a randomized controlled crossover trial. J Clin Med. (2021) 10:2880. doi: 10.3390/jcm10132880, PMID: 34209581 PMC8269272

[86] Eskandari D Zou D Grote L Hoff E Hedner J. Acetazolamide reduces blood pressure and sleep-disordered breathing in patients with hypertension and obstructive sleep apnea: a randomized controlled trial. J Clin Sleep Med. (2018) 14:309–17. doi: 10.5664/jcsm.6968, PMID: 29510792 PMC5837832

[87] Revol B Jullian-Desayes I Bailly S Tamisier R Grillet Y Sapène M. Who may benefit from diuretics in OSA?: a propensity score-match observational study. Chest. (2020) 158:359–64. doi: 10.1016/j.chest.2020.01.050, PMID: 32119859

[88] Joosten SA Tan M Wong A-M Landry SA Leong P Sand SA. A randomized controlled trial of oxygen therapy for patients who do not respond to upper airway surgery for obstructive sleep apnea. J Clin Sleep Med. (2021) 17:445–52. doi: 10.5664/jcsm.8920, PMID: 33094725 PMC7927316

[89] Oksenberg AS. Positional therapy for sleep apnea: a promising behavioral therapeutic option still waiting for qualified studies. Sleep Med Rev. (2014) 18:3–5. doi: 10.1016/j.smrv.2013.08.003, PMID: 24100064

[90] Ong JC Crawford MR Dawson SC Fogg LF Turner AD Wyatt JK. A randomized controlled trial of CBT-I and PAP for obstructive sleep apnea and comorbid insomnia: main outcomes from the MATRICS study. Sleep. (2020) 43:zsaa041. doi: 10.1093/sleep/zsaa041, PMID: 32170307 PMC7487869

[91] Tu AY Crawford MR Dawson SC Fogg LF Turner AD Wyatt JK. A randomized controlled trial of cognitive behavioral therapy for insomnia and PAP for obstructive sleep apnea and comorbid insomnia: effects on nocturnal sleep and daytime performance. J Clin Sleep Med. (2022) 18:789–800. doi: 10.5664/jcsm.9696, PMID: 34648425 PMC8883096

[92] McNicholas WT Bassetti CL Ferini-Strambi L Pepin JL Pevernagie D Verbraecken J. Challenges in obstructive sleep apnoea. Lancet Respir Med. (2018) 6:170–2. doi: 10.1016/S2213-2600(18)30059-6 29428843

[93] Gauld C Micoulaud-Franchi JA. Why could sleep medicine never do without polysomnography? J Sleep Res. (2021) 31:e13541. doi: 10.1111/jsr.13541 34927296

[94] Lim DC Mazzotti DR Sutherland K Mindel JW Kim J Cistulli PA. Reinventing polysomnography in the age of precision medicine. Sleep Med Rev. (2020) 52:101313. doi: 10.1016/j.smrv.2020.101313, PMID: 32289733 PMC7351609

[95] Pepin JL Bailly S Tamisier R. Incorporating polysomnography into obstructive sleep apnoea phenotyping: moving towards personalised medicine for OSA. Thorax. (2018) 73:409–11. doi: 10.1136/thoraxjnl-2017-210943 29477990

[96] Lechat B Hirotsu C Appleton S Younes M Adams RJ Vakulin A. A novel EEG marker predicts perceived SLEEPINESS AND poor sleep quality. Sleep. (2022) 45:zsac051. doi: 10.1093/sleep/zsac051, PMID: 35554584

[97] Bonsignore MR Suarez Giron MC Marrone O Castrogiovanni A Montserrat JM. Personalised medicine in sleep respiratory disorders: focus on obstructive sleep apnoea diagnosis and treatment. Eur Respir Rev. (2017) 26:170069. doi: 10.1183/16000617.0069-2017 29070581 PMC9489118

[98] Pien GW Ye L Keenan BT Maislin G Bjornsdottir E Arnardottir ES. Changing faces of obstructive sleep apnea: treatment effects by cluster designation in the Icelandic sleep apnea cohort. Sleep. (2018) 41:zsx201. doi: 10.1093/sleep/zsx201, PMID: 29301021 PMC5914389

